# Editorial: Neuropsychology of human growth

**DOI:** 10.3389/fpsyg.2024.1536678

**Published:** 2024-12-16

**Authors:** J. V. Orón Semper, Samuel Asensio, Francisco Ceric, Manuel E. Cortés, Inmaculada Lizasoain

**Affiliations:** ^1^Unidad de Educación Médica, Universidad Francisco de Vitoria, Madrid, Spain; ^2^Departamento de Ciencias Biomédicas, Universidad Centro de Estudios Universitarios (CEU) Cardenal Herrera, Valencia, Spain; ^3^Laboratorio de Neurociencia Afectiva, Universidad del Desarrollo, Santiago, Chile; ^4^Dirección de Investigación, Universidad Bernardo O'Higgins, Santiago, Chile; ^5^Institute for Advanced Materials and Mathematics, Public University of Navarre, Pamplona, Spain

**Keywords:** human growth, human development, applied psychology, neuropsychology, integrality

The issue of human growth has been approached from many different disciplines, such as philosophy, psychology or anthropology. The diverse perspectives adopted by researchers as renowned as Piaget, Kohlberg, Erikson, Freud, or Whitehead range from the characterization of human growth as the ability to cope with the complexity of reality (Piaget, 1965) to regarding growth as a greater integration of different human facets (Kohlberg, 1981; Cook-Greuter, 2000; Polo, 2015) or an intensification of the relational experience (Whitehead, 1978; Polo, 2015).

The publications in the present Research Topic broaden the interdisciplinary view of the issue of human growth by adding the fields of neuroscience and applied psychology. This new approach is expected to facilitate a deeper understanding of the processes associated with human growth which could lead to an improvement of wellbeing and the achievement of a fulfilling life.

In most of these papers, human growth is regarded as synonymous with psychosocial development. For instance, the study carried out by Crespo-Eguílaz et al. analyses, in the case of athletes with intellectual disabilities, challenges such as autonomy, competence and relatedness as enhancers and indicators of growth by assessing the impact of the “Más Que Tenis” (More Than Just Tennis) inclusive recreational sports programme. The findings reveal a positive correlation between the participation in such an inclusive environment and improvements in physical skills, social integration and interpersonal relationships. This research highlights how an appropriate environment can be essential to foster human growth.

The benefits of a supportive environment for personal growth are also reflected in the study by Lu et al., which explores the influence of family and school socioeconomic status (SES) on aggressive behavior in adolescents. The research shows that when family and school SES are congruent, the levels of aggression are significantly lower, while SES discrepancy (family-school) increases the likelihood of aggressive behavior in adolescents. In addition, a positive parent-child relationship acts as a buffer in unfavorable SES contexts, which suggests that both parental support and SES congruence are not only related to behavior, but also to personal development.

del Río et al. highlight that personal growth is nurtured not only by emotional stability but also by that personal growth is nurtured not only by emotional stability but also by hormonal balance, especially during periods of significant change such as adolescence. By investigating how ovulatory dysfunctions in Chilean adolescents affect personal growth and psychological wellbeing, the authors show that altered levels of neuroactive hormones, such as free testosterone and estradiol, are associated with mood and self-esteem, both of these being regarded as key elements for personal growth. Participants with hormonal dysfunction showed elevated levels of depression, confusion and fatigue, which suggests a significant interplay between biological and psychological factors during adolescent development.

All these papers seem to point to a close relationship between human growth and a greater integration of the person both ad intra and ad extra. For instance, the work of Zhao et al. analyses the impact of a significant crisis on academic control and academic emotions among students who have coped with a natural disaster. The research identifies a complex relationship between post-traumatic stress disorder and perceived academic control, showing that students who developed a positive sense of academic control experienced less anxiety and greater academic wellbeing. By applying Pekrun's control-value theory, perceived control is suggested to be crucial for the development of positive emotions and, ultimately, for the restoration of learning in adverse situations. Students' ability to manage their academic emotions and establish a sense of positive control after a traumatic event is essential for their psychological growth and academic success, which reinforce the observation that educational environments may play a crucial role in mitigating negative effects of traumatic experiences and fostering long-term wellbeing.

Bernacer examines human growth from an evolutionary perspective, concluding that caring for vulnerable individuals is essential for the survival and social and cognitive development of the human species. As such, Bernacer does not only place this capacity to care for and support the most vulnerable in relation to caregivers' personal growth, but also in relation to a gain in social cohesion, which gives a sense of purpose and strengthens the social fabric. In this sense, the act of caring is itself a source of personal growth and social development, which underlines the importance of human relationships in individual and collective progress.

Taken together, these studies shed light on the complexity of personal growth from a neuropsychological and social perspective, embracing issues as apparently far apart as the satisfaction of psychological needs in inclusive contexts or the role of social and biological support in adolescent development. Furthermore, the centrality of integration suggests that no biological or psychological need can be fully understood separately from the whole; such understanding requiring changing the paradigm from focusing on psychology alone or biology alone to consider the whole person, who is more than biology and psychology. Interdisciplinary dialogue is thus urged. Future research might focus on a deeper understanding of the interaction between all relevant factors over the long term in diverse cultural contexts, in order to develop intervention strategies which go beyond promoting wellbeing and resilience—both in individuals and collectives—to foster human growth. The dialogue between neuroscience and applied psychology in the study of such an anthropological issue as personal growth, allows for a richer and more multidimensional understanding of the human being, providing a comprehensive framework for future research in this emerging field. These studies highlight the need for interventions oriented toward personal growth that, within an anthropological framework, simultaneously consider hormonal, psychological, cultural, and personal factors among others. This effort to integrate all human dimensions can enrich many different disciplines, such as education (Orón Semper, 2023).
